# District health executives in Midlands province, Zimbabwe: are they performing as expected?

**DOI:** 10.1186/1472-6963-12-335

**Published:** 2012-09-22

**Authors:** Mary Muchekeza, Anderson Chimusoro, Notion T Gombe

**Affiliations:** 1 Field Epidemiology Training Program, University of Zimbabwe, P O Box A178, Avondale, Harare, Zimbabwe; 2 Provincial Medical Directorate, Midlands Province, Ministry of Health and Child Welfare, Harare, Zimbabwe

**Keywords:** District health executives, Performance, Midlands Province

## Abstract

**Background:**

The cornerstone of the health system in Zimbabwe, the district health system has been under the responsibility of the district health executive since 1984. Preliminary information obtained from some provincial health managers in Midlands Province suggested a poor performance by most district health executives. We therefore investigated the reasons for this poor performance.

**Methods:**

A descriptive cross sectional study was conducted. Structured interviewer administered questionnaires were used to obtain information from district health managers of five randomly selected districts in the province. Checklists were used to assess resource availability, staffing levels and proxy indicators to effective district health executive function. Data were analysed using Epi Info statistical package.

**Results:**

Thirty district health managers were interviewed. Almost half of the participants could not list at least five functions of district health executives. Twenty nine managers reported having inadequate management skills requiring training. District health executives failed to meet their targets on expected activities in the year 2010 such as conducting monthly district health executive meetings, conducting quarterly supervision to health centres and submitting quarterly district health reports to the provincial level.

**Conclusion:**

Poor knowledge on expected functions could have resulted in poor performance. Without adequate management training district health managers are likely to underperform their duties. DHE guidelines were therefore distributed to all districts. Management trainings were conducted to all district health executives throughout the country from November 2011.

## Introduction

Since the Alma Ata declaration in 1978, Primary Health Care (PHC) remains the core issue in the health delivery systems of most African countries including Zimbabwe. [1].

The district health system is the cornerstone of the health system in Zimbabwe [2]. Health facilities within a health district are under the responsibility of the District Health Executive (DHE). This team comprises of the District Medical Officer, District Nursing Officer, District Environmental Health Officer, District Health Services Administrator, pharmacist and accountant. The DHE is responsible for planning and coordinating all district health activities [3,4].

The main objective of establishing the DHE is to enable decentralization of administrative functions to the districts. This entails regular meetings by district health managers, supervision of clinics within the districts, authority over financing and operations of the health services [5].

Preliminary information obtained from some Provincial Health Executive (PHE) members in Midlands Province in 2011 suggested a poor performance by most DHEs. This study therefore sought to assess the performance of District Health Executives in the province and to identify the possible reasons for their poor performance.

## Materials and methods

A descriptive cross sectional study was conducted in Midlands Province health districts among district health services managers. The Provincial Environmental Health Officer (PEHO), Provincial Nursing Officer (PNO) and Provincial Health Services Administrator (PHSA) were interviewed as key informants.

**Figure 1:**
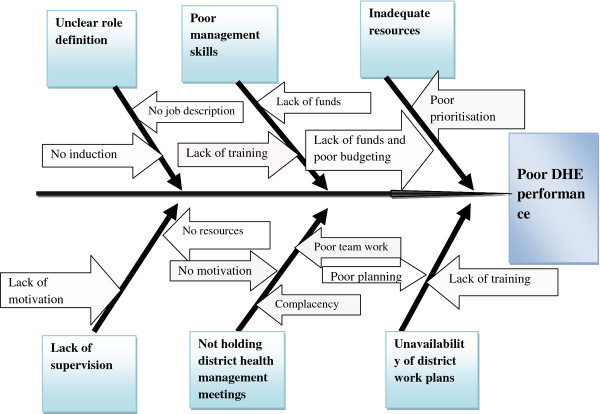
Ishikawa analysis for poor DHE performance in Midlands province, 2011.

Five out of the eight districts in Midlands province were randomly selected. The selected districts were Gokwe south, Chirumanzu, Gweru, Shurugwi and Zvishavane. All district health managers from the selected districts participated in the study. Interviewer administered questionnaires drafted with the guidance of the root cause analysis diagram (Ishikawa) [6] were used to obtain demographic information and information on job descriptions, supervision, planning, management meetings, management training and resource availability from health managers at district level. The Ishikawa diagram used is shown in Figure1. 

Checklists were used to assess resource availability for DHEs for the year 2010, number of DHE meetings, weekly disease surveillance meetings and collaborative meetings held in the district in the year 2010.

Quantitative data generated from questionnaires was captured, processed and analyzed using Epi –info version 3.5.1 statistical package. Variables were summarized and reported as frequencies and proportions. Data generated from open ended questions in the questionnaire was sorted and analyzed for content. Summaries were presented as proportions and frequencies of responses reported that fell under differing categories

Permission to carry out the study was sought from the Health studies office, the Provincial Medical Director Midlands Province and the District Medical Officers of the selected districts. Written consent was obtained from all study participants. Confidentiality was assured and maintained throughout the study.

## Results

A total of 30 district health managers were interviewed. The majority (76.7%) of them were male. Their median age was 35 years (Q_1_ = 32; Q_3_ = 50), median years in service were 9.5 years (Q_1_ =6; Q_3_ = 15) and their median number of years in the current management post was 4.5 years (Q_1_ = 3; Q_3_ = 6). There were five participants in each of the designations: District Medical officers, District Nursing Officers, District Environmental health officers, District health services administrators, Accountants and Pharmacists.

Three key informants were interviewed namely the Provincial Environmental Health Officer, the Provincial Nursing Officer and the Provincial Health Services Administrator. Two of the key informants were female while one was male. Their median age was 49 years (Q_1_ = 41; Q_3_ = 55). Their mean number of years in service was 25 years (standard deviation =9). The median number of years in current management post held was 5 years (Q_1_ = 3; Q_3_ = 8).

None of the district health management posts were fully filled with substantive managers. The worst were District Medical Officer Posts which were only 25% substantively filled. Shurugwi, Zvishavane, Chirumanzu and Gokwe south districts were all headed by none substantive district medical officers.

Most(93.3%) of the district managers exhibited good knowledge on the number of posts making up the DHE, who constitutes the DHE, who chairs the DHE, their individual roles as DHE members and the expected number of DHE meetings to be held in a year. Knowledge on the functions of the DHE was however lacking since a significant proportion of the participants could not list at least five functions of the DHE as shown in Table1.

**Table 1:** Knowledge of DHE roles and functions among district health managers in Midlands Province, 2011

**Variable**	**Number of managers correctly reporting variable** **n = 30 (%)**
Number of posts making up DHE	28 (93.3)
Knowledge on who constitutes the DHE	28 (93.3)
Knowledge on who chairs DHE	29 (96.7)
Knowledge of at least 5 functions of DHEs	16 (53.3)
Aware of individual role as DHE member	30 (100)
Knowledge on frequency of DHE meetings/year	30 (100)

The median number of district executive meetings held in the year 2010 that were reported by the district health managers was 5 (Q1 = 4; Q3 = 6). The worst performing districts in this regard were Gweru and Zvishavane each having held three and two DHE meetings in the year 2010 respectively. Shurugwi and Gokwe south were the better performing districts as shown by their having held more DHE meetings and having conducted supportive supervisory visits to their health centres.

Most DHEs failed to meet the targets on their expected functions and activities such as conducting district health management meetings and supervision to rural health centres as shown in Table2.

**Table 2:** DHE functions and proxy indicators for effective function of DHEs in Midlands Province, 2010

**Activity**	**Target/year**	**Target achieved**				
		**Zvishavane**	**Gweru**	**Shurugwi**	**Chirumanzu**	**Gokwe south**
DHE meetings	12	2	3	10	5	8
Supervision to each health centre	At least 3	2	1	3	2	2
DHT meetings	3	0	0	1	1	1
Submission of quarterly reports	3 reports	2	2	2	3	3
Disease surveillance meetings	52	5	5	30	24	38
Consolidated work plans available	Yes	Yes	No	No	Yes	Yes

Consolidated district work plans were availed for the researcher to see in three out of the five visited districts namely Zvishavane, Chirumanzu and Gokwe south. The reasons given for failure to achieve 2010 plans included inadequate resources, poor team work, lack of motivation and lack of time.

In the year 2010 resources such as human resources, vehicles, funds, printers and stationary were reported by all districts to have been inadequate.

Eleven (36.7%) of the interviewed district health managers reported having been given job descriptions before assuming duties relevant to their current management post. However five out of the 11 managers had a copy of their job descriptions readily available. The majority (66.7%) of interviewed health managers reported having experienced conflicts due to unclear roles.

Provincial managers indicated that some of the district managers were inducted onto their jobs but all could not enumerate the proportion of all district managers who had been inducted. The main reason given for failure to induct district managers were lack of time on the part of provincial managers, rapid staff turnovers requiring frequent inductions which have not been feasible and general neglect of this management issue.

**Figure 2:**
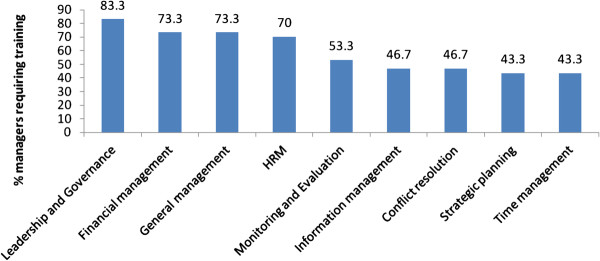
Management training needs for district health managers in Midlands Province, 2011.

Almost all (29/30) district health managers interviewed reported having inadequate management skills to effectively undertake their management responsibilities. The areas that were reported as requiring training included leadership and governance, monitoring and evaluation, human resource management, strategic planning and general health services management as shown in the Figure2.

Twenty nine (96.7%) of the district managers reported that provincial supervisors did make supportive supervisory visits to the districts. Fifty eight percent (58.6%) reported that these visits were being made quarterly while 37.9% reported them being made erratically. District managers who reported supervisory visits being made quarterly were from Shurugwi, Gweru and Chirumanzu districts while those from Gokwe and Zvishavane districts reported these visits being made on an erratic and unscheduled basis.

Ninety percent (90%) of district health managers reported resources such as funds, vehicles and human resources as having been inadequate in the year 2010. Shurugwi district however reported having had adequate stationary, printers and photocopiers in the same year. None of the districts had DHE guidelines or reference material for guidance on the expected operations of DHEs.

Policy changes and guidelines were reported by two out of the three key informants not to be always communicated in time to district health managers. This was reported to be due to mailing problems and problems of interpretation and understanding of the policy issues at the provincial level before cascading it down to district level.

The majority (83.3%) of the district health managers interviewed reported that their DHEs were not performing to expected standards. The most common reported barriers to effective DHE performance were inadequate resources, lack of management training and poor team work among district health managers as shown in Table3. Resource availability, supervision and management training were rated by all district health managers as strong determinants of effective DHE function.

**Table 3:** Barriers to effective DHE performance in Midlands Province, 2011

**Barriers**	**Number of district managers reporting barrier n = 30 (%)**
Inadequate resources	24 (80)
Lack of management training	21 (70)
Poor team work	17 (56.7)
Lack of supervision	10 (33.3)
Lack of motivation	9 (30)

## Discussion

Our study has revealed that of the five sampled districts, Zvishavane and Gweru districts performed poorly compared to Gokwe south district and Shurugwi district. The poor performance could be attributed to a number of factors namely lack of management training among district health services managers, poor team work, inadequate resources, lack of induction onto the job and lack of knowledge on DHE functions among managers.

None of the district management posts were completely filled with substantive managers. This could have gravely affected execution of roles and tasks in the Province as managers in acting capacity may not seriously assume their responsibilities. The constitution of District Health Executives has a bearing on the overall provision of health care services in the district.

This is supported by a study done in Kenya which showed that district health management teams were often poorly constituted and lacked requisite capacity to undertake their function. The teams were reported to be lax, sloppy, inconsistent and ineffective [7].

Lack of knowledge on DHE functions could have resulted in district health managers underperforming their duties. This knowledge would ensure that each manager would align their plans and activities to achievement of functions of the DHE as a whole. Without this fundamental knowledge many functions or activities could have been left undone.

Although DHE members in Midlands knew the expected number of DHE meetings to be held in a year, none of the five districts had achieved the target of monthly DHE meetings in the year 2010. Failure to hold these meetings meant there was no forum to discuss district management issues and pave ways for improving provision of health services in the districts.

This is supported by *Egger et al., 2007* in their study in South Africa, Togo and Uganda where a regular forum for managers to identify their needs, discuss problems and share ideas was identified as one of the several ways to help health services managers do their jobs well [8].

The availability of district work plans in three of the five districts may have meant that the other two districts were not targeting their activities towards achievement of any goals leading to overall poor performance.

Some literature has however demonstrated that district management teams must display a clear vision of what they intend to do and must demonstrate adequate planning to ensure the highest chance of meeting their goals [9].

Lack of resources was cited by DHEs as a major barrier to their effective performance. This lack of resources could also work as a de-motivating factor within the work environment further worsening the performance of district health teams.

The lack of job descriptions and poor clarity on some tasks could have greatly undermined the potential for good performance by DHEs as some activities could have been left undone.

In order to perform well, health managers and employees in general need to know what is expected of them. A detailed job description is therefore necessary for every post within the DHE so that members are clear on what tasks they are expected to perform [10].

Lack of clarity on some tasks may also have resulted in ineffective delegation by district health managers, further undermining DHE performance. Almost all the managers reported having inadequate management skills to effectively undertake their management responsibilities. Without adequate management training, district health managers are likely to underperform their duties.

District health managers therefore need to have the technical competence to perform the tasks expected of them. Competence in health management requires skills in supervision, training and problem solving [11].

Our findings are also supported by *Filerman et al.*, 2003 who reported in their paper that health systems worldwide are facing a lack of competent management at all levels and that this is the most fundamental barrier to health resources reaching the general public. They maintained that lack of competent managers at all levels fosters “vertical” health programmes that have narrow targets and are centrally planned [12].

*Kolemainen et al., 2004* also reported that untrained health managers and weak personnel systems have been noted to be ill equipped to cope with the added complexity of decentralisation of health services [13].

Leadership and governance is a very critical management aspect for enhancing DHEs’ performance yet only a minority of DHE members in Midlands had been trained in this aspect. When the team carries out leadership tasks, a shared vision is created which allows the health system efforts to be pulled in a single direction. This is supported by *Kumar et al., 2010* who reported an increased focus on health system performance in three districts of Jharkhand, India after implementing an innovative leadership development and organisational effectiveness intervention over a period of three years [14].

In another study done in India to develop and test key management interventions in order to improve the performance, efficiency and effectiveness of primary health care delivery at Hoshangabad district, building the capacity of managers and developing their management and leadership skills were reported as crucial factors for the effective delivery of primary health care services [15].

Supervision was reported by a significant proportion of district health managers to be erratic, yet it provides a basis for discussion between district managers and their supervisors. It also provides a learning opportunity for district managers through on job training of some management aspects from provincial supervisors. This is supported by findings from a study done by *Egger et al., 2007* in South Africa, Togo and Uganda where on the job support was perceived by many managers as key to improving their performance [8].

DHE guidelines or reference material were unavailable for use in guiding district health managers to perform their duties. When compounded by delayed communication of policy changes from the provincial level, this may mean the DHEs lacked direction on what duties to perform, by whom, when and how often resulting in poor performance and failure to achieve DHE targets.

This is supported by findings from a study done by *Egger et al. 2007*, where the availability of reference books and guidelines was identified as one of the ways to help health services managers do their jobs well [8].

### Study limitations

Our main study limitation was inadequate time which led us to draw inferences from response given by the very people whose performance was being assessed. This could have introduced perception bias into this study. Being a descriptive study meant the authors mainly concentrated on reporting numbers as a measure of performance and did not go further to assess actions taken as a result of some activities having been done.

## Conclusion

Our study therefore concluded that district health executives in Midlands Province are performing poorly with Gokwe South and Shurugwi districts generally performing better than others .Lack of management training was the major contributing factor to the poor performance by DHEs in Midlands Province.

### Recommendations

The Provincial Health Executive therefore needed to provide DHE guidelines and reference material to all DHEs, train all DHEs in management, provide job descriptions and on job induction to all district health managers . Provincial supervisors were encouraged to conduct regular supervision to all districts. The district health managers needed to hold regular district management meetings to plan and discuss district health activities in line with the expectations of DHE guidelines.

### Actions taken as a result of the study

Following this study three District Medical Officers were promoted to be substantive. Management trainings were conducted throughout the country to equip District Health Executives with leadership and governance skills. District Health Executive guidelines were distributed to all districts.

## Competing interests

All authors declare no competing interests.

## Author contributions

MT provided overall technical and academic guidance, and reviewed several drafts of the article. GS was responsible for providing academic guidance and reviewed several drafts of the article. MM was the principal investigator responsible for developing the research protocol, designing the data collection tool, collecting the data and compiling the final study report. AC was responsible for providing technical support to the principal investigator during field work. NG was responsible for providing academic guidance and reviews of the study protocol, data collection tools, the report and the final manuscript. All authors read and approved the final manuscript.

## Pre-publication history

The pre-publication history for this paper can be accessed here:

http://www.biomedcentral.com/1472-6963/12/335/prepub
